# Puerperal Convulsions

**Published:** 1890-10

**Authors:** W. A. Yingling

**Affiliations:** Nonchalanta, Kansas


### PUERPERAL CONVULSIONS.—A CASE.

W. A. Yingling, M. D., Nonchalanta, Kansas.

On January 20th, 1889, at twelve, noon, I was called to wait upon Mrs. G., a strong, apparently healthy and vigorous woman, of close, firm fibre; saucy, lively, and determined to bear her ordeal without fear or murmur. Being a primipara I expected a tedious, slow case, but at the commencement I had no idea I should have a case occurring but once in more than five hundred parturitions. I found her more or less anxious and uneasy, yet heroically laughing and joking to keep up her courage. Two old ladies were present as assistants, neither versed in the science of midwifery, and ignorant of everything professional except "teas and yarbs."

Upon examination I found the parts dilating, but was unable to reach the child. I apprehended tediousness, but kept my own counsel, concluding to do the best thing possible, to wait. I could not determine a transverse presentation. Whilst waiting, as is my custom, I refreshed my memory on all possible contingencies. After several hours, the pains coming every fifteen or twenty minutes, I made another examination and found I could just reach the head of the child. The presentation was normal except face upward toward the pubes. Progress was very slow; patient uneasy, but confident; the bag of waters normal; the assistants urging teas and relating yarns; myself anxious and foreboding, yet cheerful. I administered *Gelsemium*, from indications, but to no use, yet the pains were some stronger. About one o'clock the next morning, after thirteen hours of labor pains, I found the child had entered the lower straits and I hoped for a speedy termination of the case. The patient was strong, courageous, and still hopeful, for she had the utmost confidence in her attending physician. I noticed about two A. M. that she was inclined to be drowsy, and began to talk foolishly, especially in calling upon her attendants, with a silly expression; her face flushed; quite nervous and anxious. I gave her a dose of *Cimicifuga-race.*, which seemed to quiet her somewhat.

I was twenty-five miles from any other physician; help was impossible. I had no instruments with me, for I had never, in all my practice, had occasion to use them. I always endeavor to assist nature, *wait*, and have found the indicated remedy sufficient *in every case* without the use of instruments, except this one. I know many homœopathic physicians claim the remedies are inadequate to meet severe cases, and that adjuncts and instruments are absolutely necessary. But I find such non-homœopathic adjuncts are useless, and instruments are needful in the very exceptional cases. I have never lost an obstetrical case out of *very* many, and have never used anything but homœopathic remedies. This case proves the homœopathic weakling in error, and that "little pills" have and can cure the severest case of puerperal convulsions.

At five A. M., I noticed the muscles of the face and mouth twitching; the eyes gradually rolling back, tremulous and dilated; the head drawing to the left and backward; foaming at the mouth; general muscular twitching. The crisis had come. The attendants in consternation and helpless. I am glad to say that in this trying ordeal I was cool and self-possessed. I at once called for a spoon to save the tongue, and placed the cooler attendant at the head to use it to the best advantage. I placed a drop of *Bell.*$^{3x}$ on the tongue during the spasm. The spasm was very severe, lasting several minutes. When she came out of the spasm she was bewildered, without knowledge of the occurrence of the convulsion. The pains held off for some time, but finally came, and with them another convulsion, but not so severe nor as long as the first one. I gave another dose of *Bell.*$^{3x}$.

It was impossible for me to remove the child by hand, though I tried at different times. On the first appearance of the crisis I had dispatched a runner for instruments; distance, five miles. During the absence of the runner the *Bell.* had controlled the case, so that only three spasms had occurred, each lighter. Being so long time in labor, and the case so severe, without any assisting physician, and concluding the child must be dead, I determined to perform craniotomy.

I first, however, applied the forceps and found that I could not remove the child in that way without danger to the mother. During the operation the patient had a very severe and long continued spasm, but I hastened as much as possible and delivered her of a very large girl baby, dead, of course. I administered another dose of *Bell.*$^{3x}$ on the tongue. She had but one more spasm, a light one, making five in all. *Bell.* was the only remedy given, and controlled the case. To add to the fear of the friends, a very profuse and bright red hemorrhage set in, with pallor of the face. I could not get subjective symptoms, but had to prescribe from the appearance of the flow. One *single* dose of *Ipecac*$^{3x}$ *at once* checked the flow without a recurrence.

The patient progressed finely, without knowledge of the events, nor of the child (until told afterward), and in ten or twelve days was up and around, apparently as well as if she had had a natural labor. I expect another call to the same lady soon.

This case proves that Homœopathy *can* cure such severe cases. I am sorry I did not have the higher potency to try, but I am just beginning to see the value of them, yet not using them extensively. I may yet become a high-potency man, for I am *honest*, and will investigate. I am on the fence with one leg over on the side of high potencies, and body leaning that way.

---

